# High-resolution temporal and regional mapping of *MAPT* expression and splicing in human brain development

**DOI:** 10.1371/journal.pone.0195771

**Published:** 2018-04-10

**Authors:** Marco M. Hefti, Kurt Farrell, SoongHo Kim, Kathryn R. Bowles, Mary E. Fowkes, Towfique Raj, John F. Crary

**Affiliations:** 1 Department of Pathology, Icahn School of Medicine at Mount Sinai, New York, NY, United States of America; 2 Department of Neuroscience, Icahn School of Medicine at Mount Sinai, New York, NY, United States of America; 3 Friedman Brain Institute, Icahn School of Medicine at Mount Sinai, New York, NY, United States of America; 4 Ronald M. Loeb Center for Alzheimer’s Disease, Icahn School of Medicine at Mount Sinai, New York, NY, United States of America; 5 Department of Genetics and Genome Sciences, Icahn School of Medicine at Mount Sinai, New York, NY, United States of America; International Centre for Genetic Engineering and Biotechnology, ITALY

## Abstract

The microtubule associated protein tau plays a critical role in the pathogenesis of neurodegenerative disease. Recent studies suggest that tau also plays a role in disorders of neuronal connectivity, including epilepsy and post-traumatic stress disorder. Animal studies have shown that the MAPT gene, which codes for the tau protein, undergoes complex pre-mRNA alternative splicing to produce multiple isoforms during brain development. Human data, particularly on temporal and regional variation in tau splicing during development are however lacking. In this study, we present the first detailed examination of the temporal and regional sequence of MAPT alternative splicing in the developing human brain. We used a novel computational analysis of large transcriptomic datasets (total n = 502 patients), quantitative polymerase chain reaction (qPCR) and western blotting to examine tau expression and splicing in post-mortem human fetal, pediatric and adult brains. We found that MAPT exons 2 and 10 undergo abrupt shifts in expression during the perinatal period that are unique in the canonical human microtubule-associated protein family, while exon 3 showed small but significant temporal variation. Tau isoform expression may be a marker of neuronal maturation, temporally correlated with the onset of axonal growth. Immature brain regions such as the ganglionic eminence and rhombic lip had very low tau expression, but within more mature regions, there was little variation in tau expression or splicing. We thus demonstrate an abrupt, evolutionarily conserved shift in tau isoform expression during the human perinatal period that may be due to tau expression in maturing neurons. Alternative splicing of the *MAPT* pre-mRNA may play a vital role in normal brain development across multiple species and provides a basis for future investigations into the developmental and pathological functions of the tau protein.

## Introduction

Alternative splicing of pre-mRNAs enables an exponential increase in phenotypic diversity without corresponding increases in genome size and plays a particularly important role in the highly complex development of the vertebrate brain [1–9]. Splicing defects have been associated with specific neuronal phenotypes, including fronto-temporal lobar degeneration (FTLD) in patients with *MAPT* splice-site mutations [10, 11], spinal muscle atrophy (SMA) [12] or Taybi-Linder syndrome [13].

The microtubule associated protein tau is a highly abundant multifunctional brain protein that undergoes alternative splicing. Tau regulates the stability of microtubules, which play a key role in axonal growth and guidance [14, 15]. It is best known for its role in neurodegenerative tauopathies such as primary age related tauopathy (PART) and Alzheimer’s disease [16]. Intriguingly a subset of tauopathies, including corticobasal degeneration (CBD), progressive supranuclear palsy (PSP), fronto-temporal lobar degeneration with tau mutations (FTLD-tau) and myotonic dystrophy, are thought to be driven by changes in *MAPT* pre-mRNA alterative splicing [17]. However, the precise mechanism whereby these changes in splicing occur and cause neurotoxicity remain unclear [18].

**Fig 1:**
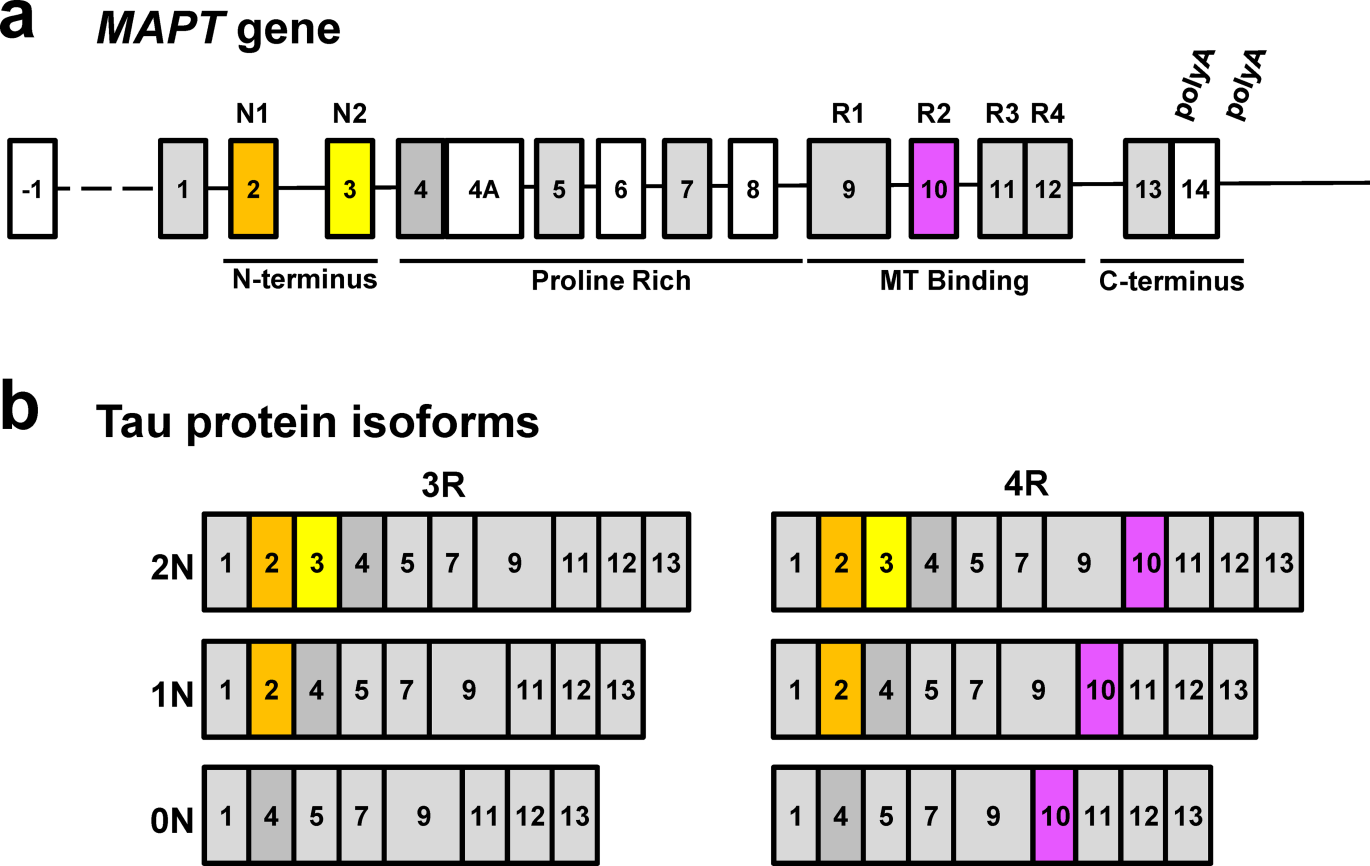
Structure of the *MAPT* gene and protein. Alternative splicing of *MAPT* produces six canonical isoforms. grey = constitutive exons, white = not expressed in human central nervous system.

Multiple tau protein isoforms exist as a result of alternative splicing of the *MAPT* gene. The gene is thought to consist of 15 exons with alternative splicing of exons 2, 3 and 10 in the central nervous system. Exons 2 and 3 create a variable N-terminal region which can contain both exons, exon 2 only or neither (2N, 1N or 0N tau respectively). Variable inclusion of exon 10 produces tau isoforms with either 3 or 4 microtubule binding domains at the C-terminus (3R or 4R tau respectively). There are therefore six canonical tau protein isoforms in the central nervous system ranging in length from 0N3R to 2N4R (Fig 1) [19].

Although the sequence of the tau protein is strongly evolutionarily conserved, there are species-specific differences in exon utilization. Adult humans have approximately equal levels of 4R and 3R tau, while rodents express exclusively 4R during adult life. Exon 8 is found in the bovine, but not human or mouse central nervous system while chickens appear to have up to 5 microtubule binding repeats (5R tau) [20–22].

Intriguingly, tau alternative splicing shifts from short to long isoforms during normal brain development in all vertebrate species studied to date, including mouse, rat, guinea pig, human and even chicken [21, 23]. Shorter isoforms have decreased microtubule binding affinity, suggesting that their expression in fetal life may allow greater neuronal plasticity [24]. This remains speculative however, since humans and mice with mutations affecting exon 10 inclusion show predominantly age-related neurodegenerative rather than developmental phenotypes [11, 25]. Understanding the role of this key protein is limited by the lack of detailed regional and temporal data on splicing changes during the prolonged and complex development of the human central nervous system. The existing human data is based on small studies with very limited sample sizes [22, 26]. Even the precise timing, duration, and neuroanatomic location of this switch in humans is largely unknown.

We therefore present, to our knowledge, the first detailed examination of the temporal sequence, neuroanatomical location and regulation of developmental changes in human tau splicing using a combination of transcriptomic datasets, quantitative PCR (qPCR) and western blotting.

## Materials and methods

### Patient samples and datasets

The patient cohort used in this paper includes multiple large publicly available de-identified transcriptomic datasets. These datasets consist of a total of 502 patients with a total of 3351 individual specimens ranging from 5.7 post conceptual weeks to 84 years. The microarray data are available in the NIH Gene Expression Omnibus (GEO) as GSE25219 (Yale) [27], GSE30272 (Lieber) [28, 29] and GSE60863 (UK/NABE) [30], while the RNA-sequencing data is available from BrainSpan [31]. The UK/NABE data do not include any cases below the age of sixteen years. Quality control and normalization was performed on each dataset by the original authors using different mathematical methods including robust multi-array average (RMA) normalization [27], Standardization and Normalization of MicroArray Data (SNOMAD)(29), the RSEQtools framework [31] and cubic spline normalization [32]. All of these methods are independent of specific housekeeping genes.

Based on publicly available information, some specimens in the BrainSpan RNA sequencing data overlap with those in the Yale dataset, but the number of shared specimens cannot be determined from the publicly available data. The datasets are otherwise independent. qPCR and western blotting were performed using snap-frozen autopsy tissue accrued under IRB-approved protocols in our laboratory. Cases with any CNS malformation or neuropathologic evidence of hypoxic-ischemic injury were excluded. A total of fourteen samples of superior fronto-parietal cortex were used for qPCR, seven fetal and seven adult. The five cases in each age group with the best preservation (as quantified by RIN score) were used for western blotting. This portion of the study was approved by the Mount Sinai Institutional Review Board (Protocol IRB-17-01313) with a waiver of informed consent. All methods were carried out in accordance with the relevant guidelines and regulations.

### Real-time PCR

Total RNA was extracted from adult human and fetal fresh frozen cortical tissue using an QIAsymphony (Qiagen, Hilden, Germany) automated RNA extraction robot according to the manufacturer’s instructions. The RNA concentration of each sample was determined using a bioanalyzer (Agilent Genomics, Santa Clara, CA). The RNA was reverse transcribed to cDNA using the high-capacity cDNA reverse transcription kit with RNase inhibitor (Cat. 4387406 Life Technologies, NY) according to manufacturer’s instructions. Gene expression in the respective tissue was quantified using TaqMan gene expression assays (Applied Biosystems), and TaqMan Gene expression master mix for each target using the QuantStudio Real-Time PCR System (Thermo Fisher, Waltham, MA) limited to only 40 cycles. The following genes were analyzed: total tau (*MAPT*, Assay ID: Hs00902193_m1), 0N tau (*MAPT*, Assay ID: hs00902188), 1N tau, (*MAPT*, Assay ID hs00902978_m1), 2N tau (*MAPT*, Assay ID: s00902314_m1), and 4R tau (*MAPT*, Assay ID: Hs00902312_m1). All of these TaqMan assays have been previously validated and extensively tested [32–34]. GAPDH was used as a reference gene (GAPDH, Assay ID: Hs99999905_m1).

### Western blots

Fresh-frozen brain tissue was homogenized with a glass-Teflon homogenizer at 500 *rpm* in 10 volumes (wt/vol) of ice-cold tissue homogenization buffer. The buffer contained 20 mM Tris, pH 7.4, 250 mM sucrose, 1 mM EDTA, 1mM EGTA and Halt protease and phosphatase inhibitor cocktail (Thermo Fisher Scientific). For dephosphorylated samples, a buffer containing 0.5% Triton-X, 50mM Tris, pH 8.0, 1mM ZnSO_4_, 1mM MgCl_2_, Halt protease inhibitor cocktail (Thermo Fisher Scientific) and Bacterial Alkaline Phosphatase (Takara Bio) was used instead. For each sample, 10 or 30 μg of proteins were separated in 10% PROTEAN TGX Precast Gels (Bio-Rad), blotted to nitrocellulose membranes, and stained with HT7 (1:3000, Thermo Fisher Scientific) for total tau. A tau ladder (RPeptide) was used as a control. HRP-labeled secondary anti-mouse antibody (Vector Labs) was detected by Pierce ECL substrate (Thermo Fisher Scientific). To quantify and standardize protein levels without reliance on specific housekeeping proteins, total protein was detected with Amido Black as previously described (Sigma-Aldrich) [35]. Chemiluminescence was measured in an LAS-4000 Intelligent Dark Box imager (Fuji Film), and relative optical densities were determined by using AlphaEaseFC software, version 4.0.1 (Alpha Innotech), normalized to total protein loaded.

### Experimental design and statistical analysis

We used unity based normalization to the range of the entire data set to eliminate negative values where necessary. Each dataset was analyzed independently. Exon- and gene-level expression were calculated as the mean of all probes located within the given exon or gene. Splicing indices were then calculated as exon expression divided by gene expression levels. Pre- and post-natal expression were compared using Student’s t-test (*t*.*test* function in R). Expression and splicing trends with time were analyzed using generalized linear models (*glm* function in R) including age, sex and RIN score as co-variates. For the Lieber dataset we also included race as a covariate. Relative gene expression in the qPCR data was determined by normalization to GAPDH followed by the ΔΔCt method to calculate relative fold expression normalized to total tau. Any outliers were removed using the Grubbs outlier test and groups were compared using a Mann-Whitney test, which was also used for western blot band quantification. For all statistical tests, p-values less than 0.05 were considered to be statistically significant with a Bonferroni correction for multiple comparisons applied where indicated. Results are reported as mean ± standard error. All data analysis was done using R and Rstudio with the exception of the PCR data which was analyzed using Graphpad Prism. Graphs were prepared using the *ggplot2* and *pheatmap* packages in R.

## Results

### Developmental shifts in *MAPT* splicing in humans

**Fig 2:**
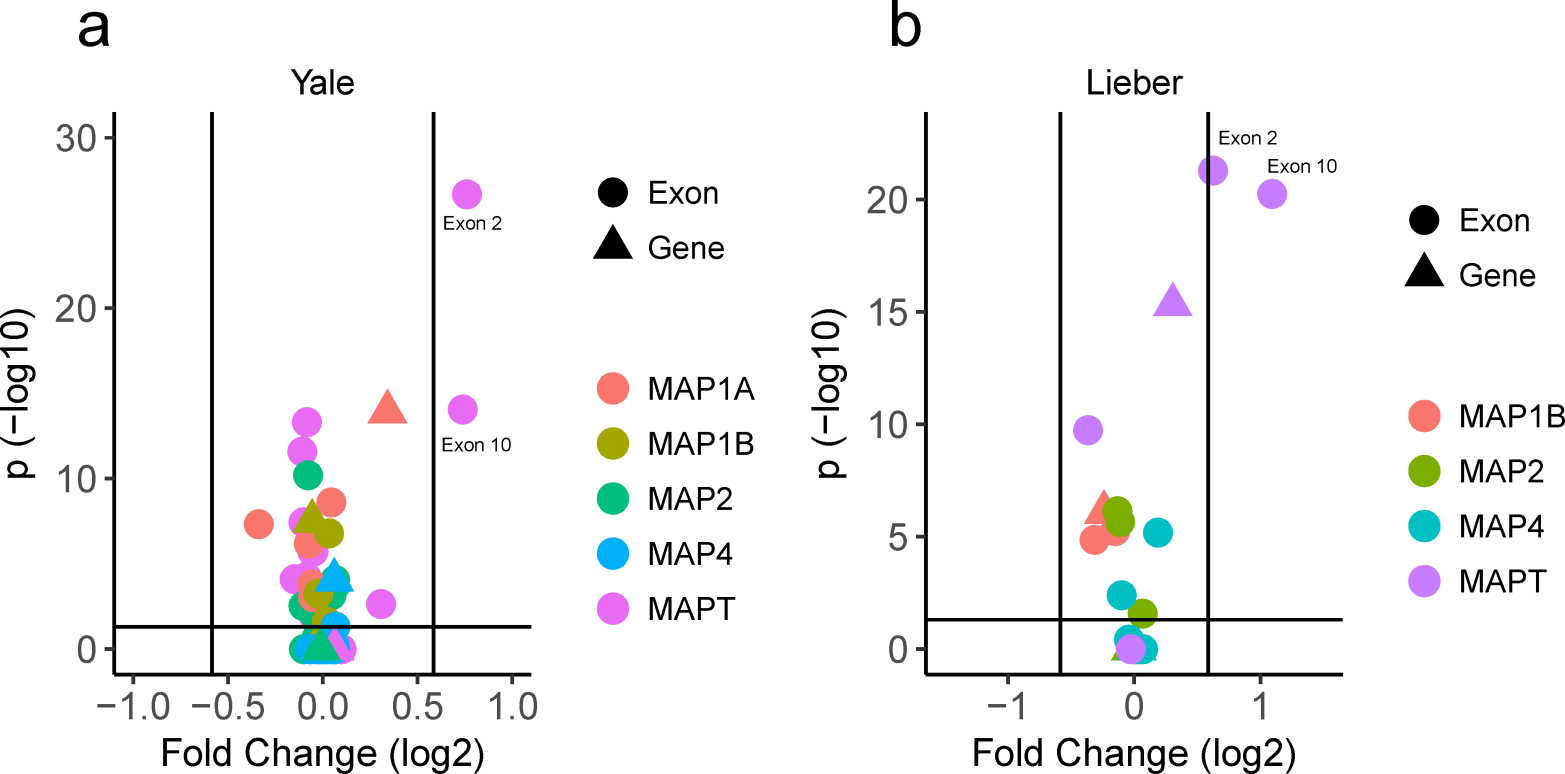
*MAPT* exons 2 and 10 show unique changes in splicing with development. Volcano plot of p-value and fold change comparing antenatal and postnatal cases by Student’s t-test using Bonferroni correction for multiple comparisons. Triangles indicate gene level data, circles exon level. Vertical line indicates 1.5-fold change, horizontal line threshold of significance.

Previous studies show developmental changes in *MAPT* exon 2, 3 and 10 utilization, but it is not clear to what degree these are also seen in other paralogous microtubule associated proteins in humans. To better understand the landscape of tau splicing during development, we began by examining *MAPT* and comparing it to all other genes and exons in the classical human microtubule associated protein (MAP) family (*MAP2*, *MAP4*, *MAP1A and MAP1B*). This revealed that in two independent data sets (Lieber, Yale), there were multiple exons and genes showing statistically significant differences between ante- and post-natal expression. Of these, only *MAPT* exons 2 and 10 showed a greater than 1.5-fold increase or decrease in expression in both data sets. Exon 2 increased 1.69 and 1.54-fold in the Yale and Lieber datasets respectively while exon 10 increased 1.66 and 2.13-fold in the same data sets (Fig 2). Of note, *MAPT* exon 3 did not demonstrate a significant change in either dataset, in contrast to animal studies showing an increase in 2N tau with age in some, but not all species studied [22, 36]. These results confirm that *MAPT* exons 2 and 10 show marked changes in splicing that are not seen in any other gene or exon in the canonical MAP family in humans.

### Abrupt perinatal transition in exon 2 and 10 expression

**Fig 3:**
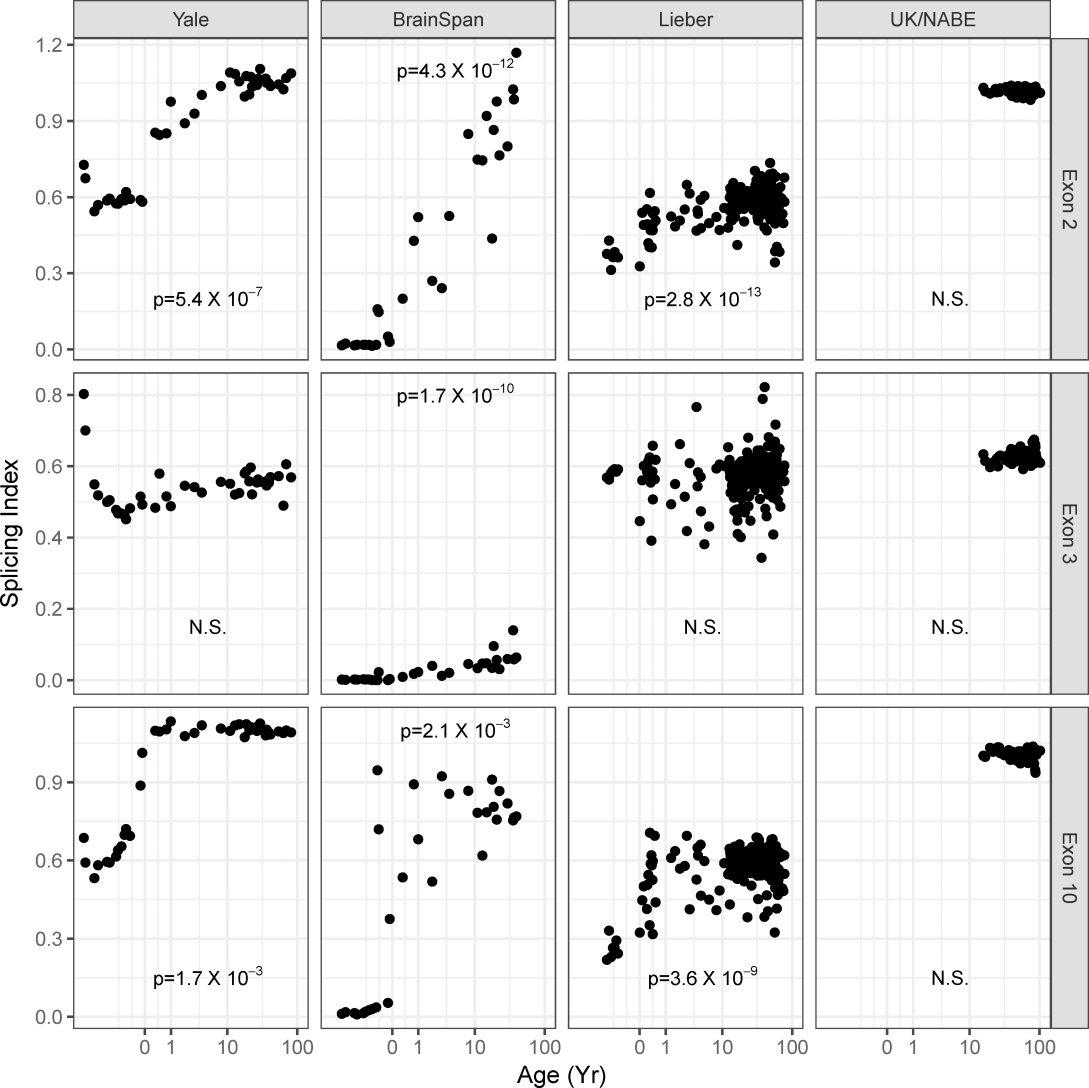
*MAPT* exons 2 and 10 show a rapid perinatal transition in alternative splicing in multiple datasets. p-values calculated using generalized linear models including available covariates within each dataset (Yale = age, sex, RIN; BrainSpan = age, sex; Lieber = age, sex, RIN, race; UK/NABE = age, sex, RIN).

We then examined the temporal sequence in exon 2 and 10 alternative splicing. Due to its key role in the N-terminal variable region of the tau protein, we retained exon 3 in our analysis as well. In all three datasets, the most dramatic shift was seen in exon 10 expression, which increased dramatically during the perinatal period, reaching a stable plateau that persisted throughout childhood and adult life (Fig 3, bottom row, left three columns). Exon 2 also undergoes a developmental transition, albeit slowly throughout childhood, reaching a plateau at an age of approximately 10 years (Fig 3, top row, left three columns). Exon 3 demonstrated a small but statistically significant change with time in the BrainSpan dataset only (p = 1.4x10^-7^) (Fig 3, center left). When we examined a fourth data set including multiple regions from 134 normal adult brains (UK/NABE) we found minimal variation in exon 2, 3 and 10 splicing during adult life from age 16 to 102 years (Fig 3, right).

**Fig 4:**
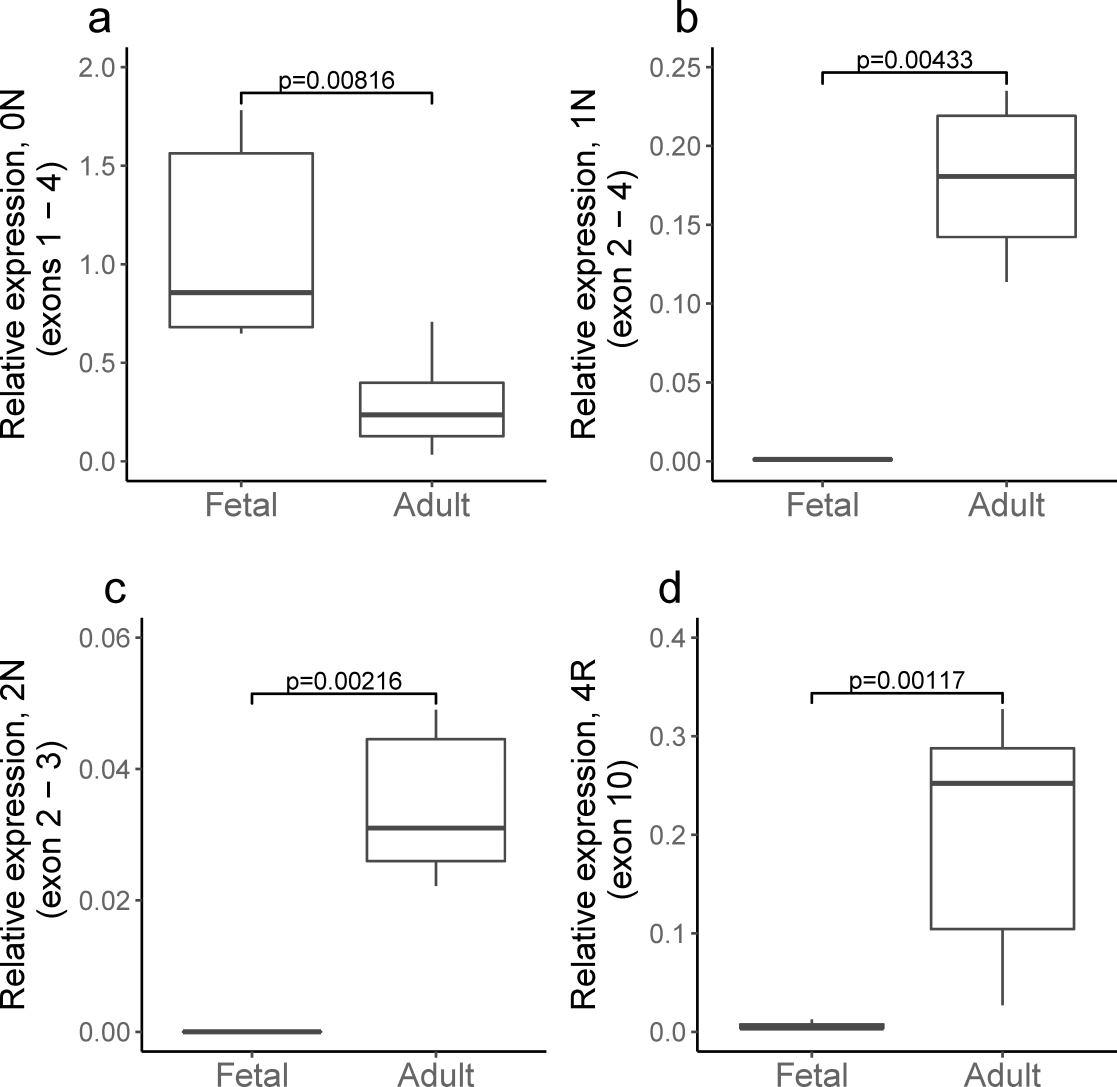
qPCR on human fetal and adult cortex demonstrating shift towards 1N and 4R tau during development. Taqman primers for **A**, 0N, **B**, 1N, **C**, 2N and, **D**, 4R tau were used with normalization to GAPDH and total tau. P-values using Mann-Whitney test.

We then used quantitative PCR to further characterize tau isoform expression in frozen autopsy brain tissue. Seven second trimester fetal cases, all superior fronto-parietal cortex, were included. Causes of death included elective termination (4), premature preterm rupture of membranes (2) and chorioamnionitis (1). Seven neurologically normal adult specimens (ages 55 to 78) were used as controls. Taqman primers specific to isoforms 0N, 1N, 2N and 4R tau showed a decrease in 0N tau (Fig 4A) with a compensatory increase in 1N tau (Fig 4B), reflecting the increased inclusion of exon 2 seen in the transcriptomic data. There was a similar, but smaller, increase in 2N tau (Fig 4C). There was a dramatic increase in 4R tau expression (Fig 4D) reflecting the increased inclusion of exon 10. Unlike the transcriptomic data where splicing index only reflects relative expression between groups, qPCR enabled us to measure the absolute exon 3 splicing index and demonstrate essentially no exon 3 inclusion in fetal brain.

**Fig 5:**
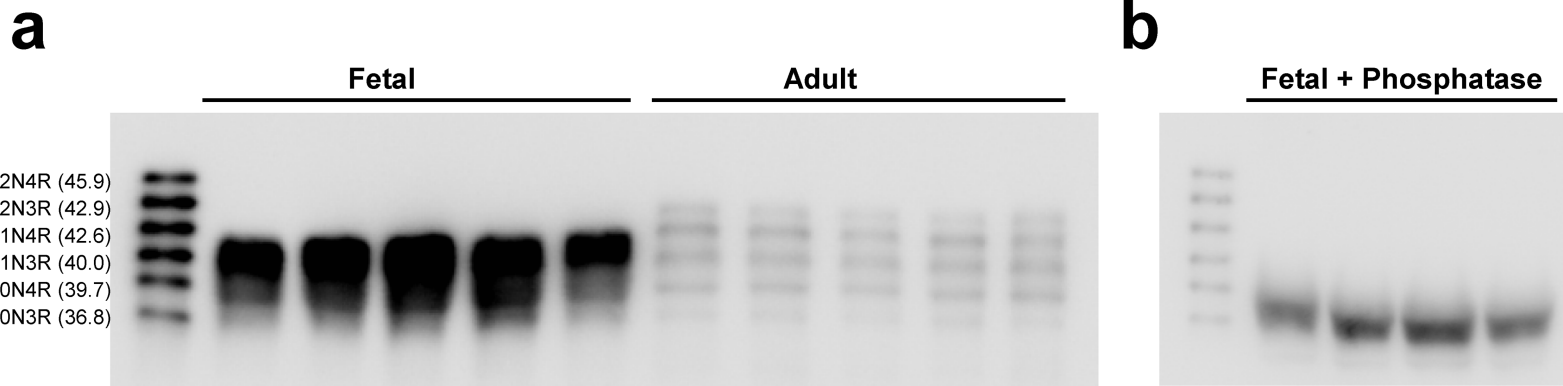
0N3R is the predominant tau proteoform in human fetal cortex. **A**, Immunoblots with fetal and adult brain homogenates were probed with antisera to total tau (HT7). **B**, Treatment of the fetal brain homogenates with phosphatase resolves the banding pattern to a single species with similar electrophoretic mobility to the 0N3R tau in the protein ladder. Molecular weights are indicated in kDa after each isoform.

To confirm these findings on the protein level, we performed Western blots on a subset of samples. Using antisera to total tau, we found fetal brain showed a complex banding pattern, whereas adult specimens showed the expected pattern of (Fig 5A). It has been reported that fetal tau is hyperphosphorylated and that this may influence the apparent molecular weight of tau [37]. We treated the fetal specimens with phosphatase, and found almost exclusively 0N3R tau (Fig 5B), which is consistent with our findings on the mRNA level.

Apart from the exon 10 probe used in the Lieber dataset, which also maps to an intergenic region on Chromosome 6, the probe sequences for exons 2 and 10 do not map to any other loci using BLAT. Neither exon 2 nor exon 10 contain any single nucleotide polymorphisms with frequency ≥1% [38]. This makes artefacts due to cross-hybridization or germline variation unlikely as a cause of the observed changes. Existing animal studies have significantly lower temporal resolution, but show a shift at an equivalent developmental stage [22]. Together, these results provide, for the first time, a detailed map of the temporal variation of tau alternative splicing during the human perinatal period.

### *MAPT* expression correlates with neuronal maturation

**Fig 6:**
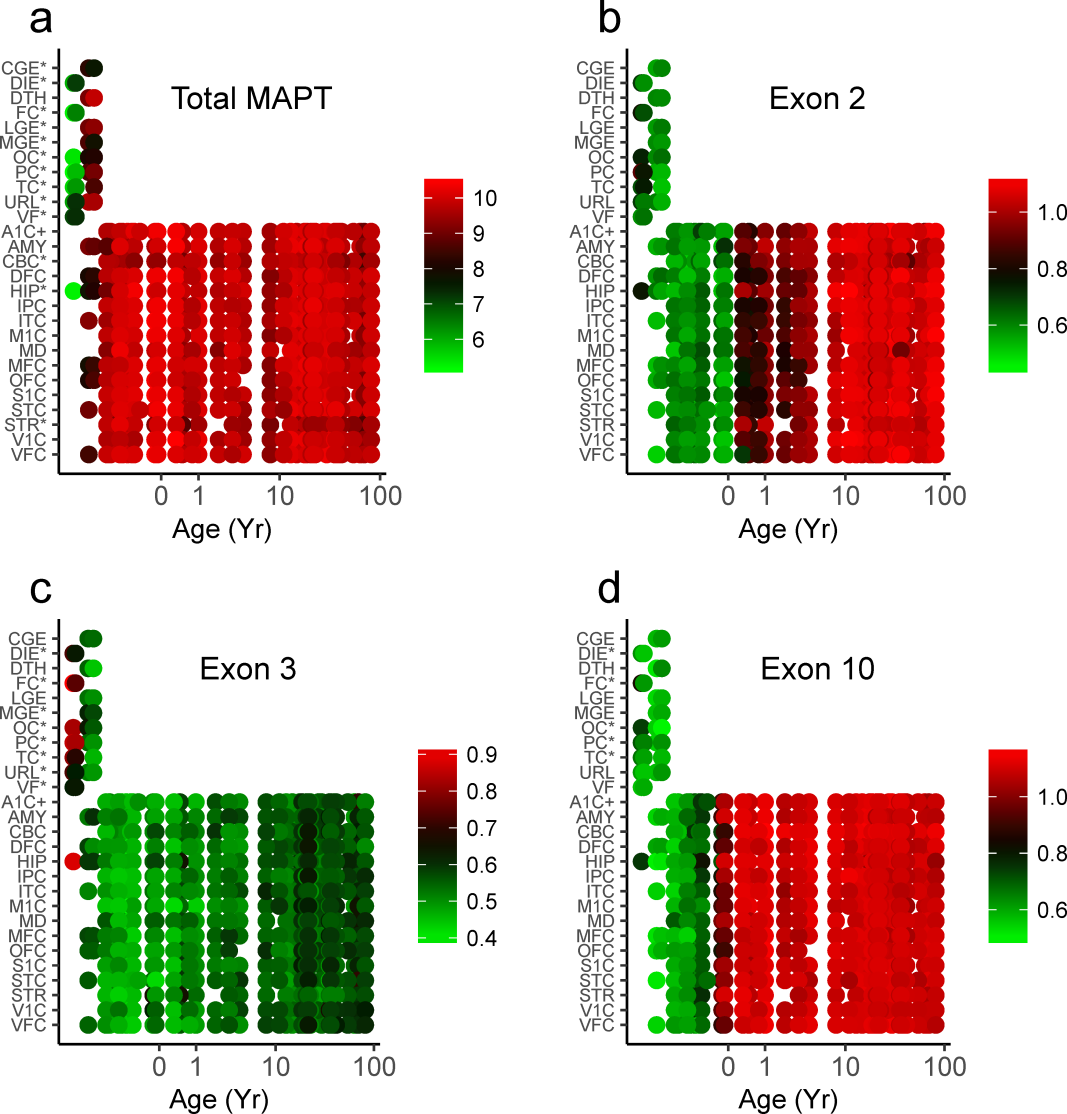
Immature regions show lower expression of *MAPT* and exon 10. ***A*,** Regional and temporal variation in *MAPT* expression, ***B*,** exon 2 splicing, ***C***, exon 3 splicing and ***D*,** exon 10 splicing. *Region with p<0.05 by generalized linear model including age, region, sex and RIN score as covariates, Bonferroni’s correction for multiple comparisons. ^+^Reference region. *CGE* caudal ganglionic eminence, *DIE* diencephalon, *DTH* dorsal thalamus, *FC* frontal cerebral wall, *LGE* lateral ganglionic eminence, *MGE* medial ganglionic eminence, *OC* occipital cerebral wall, *PC* parietal cerebral wall, *TC* temporal cerebral wall, *URL* upper rhombic lip, *VF* ventral forebrain, *A1C* primary auditory cortex, *AMY* amygdala, *CBC* cerebellar cortex, *DFC* dorsolateral prefrontal cortex, *HIP* hippocampus, *ITC* inferior temporal cortex, *M1C* primary motor cortex, *MD* mediodorsal nucleus of the thalamus, *MFC* medial prefrontal cortex, *OFC* orbital prefrontal cortex, *S1C* primary sensory cortex, *STC* superior temporal cortex, *STR* striatum, *V1C* primary visual cortex, *VFC* ventrolateral prefrontal cortex, regions are defined in [27].

Existing data on changes in tau expression and splicing is based largely on large-scale tissue homogenates and thus lacks neuroanatomical detail. We therefore examined *MAPT* expression as a function of individual brain regions sampled throughout development in the Yale dataset (Fig 6A). Overall *MAPT* expression did not vary with time but was significantly lower in neurogenic regions such as the caudal, lateral and medial ganglionic eminences (CGE, LGE, MGE), and the upper rhombic lip (URL, Fig 6A). It is also significantly lower in regions containing significant numbers of migrating immature neurons, including full-thickness sections of fetal frontal, occipital, parietal and temporal cortices (FC, OC, PC and TC) as well as the hippocampus (HIP). Exon 2 of *MAPT* showed no significant regional variation in splicing (Fig 6B). Exon 3 showed statistically significant but small increases in expression in immature areas (Fig 6C) while exon 10 showed the opposite pattern (Fig 6D). There is thus little variation in tau splicing between cortical regions, with the only significant differences seen between neurogenic regions (germinal matrix, rhombic lip) and other structures.

## Discussion

To date, studies of developmental alternative tau splicing have been largely limited to animal models, with very little data available from human brain tissue. Our data broadly supports the observed developmental shift from short to long tau isoforms during human brain development with several intriguing differences [20, 22, 24]. Interestingly, we were also able to show that fetal brain contains higher levels of phosphorylated tau protein, which confirms studies in animals [39] and in human CSF [26]. Expression of tau mRNA correlates with neuronal differentiation, being lowest in neuronal precursors of the germinal matrix and sub-ventricular zone and highest in the cortical plate, where neurons are differentiating and forming axons, a process where microtubules are critical.

Exons 2 and 10 of the *MAPT* gene show a dramatic developmental change in splicing that is unique in the human microtubule associated protein family. Even *MAP2*, evolutionarily closely related to *MAPT*, shows minimal variation in splicing. In contrast, exon 3 splicing shows relatively small, albeit significant, increases during development. Quantitative differences in splicing between datasets may be due to known technical differences between Affymetrix and Illumina platforms [40] and between microarray and RNA sequencing data [41]. Based on our data, the shift in *MAPT* alternative splicing occurs during the end of the last trimester of fetal development and the first months of post-natal life. This time period captures neuronal migration from neurogenic areas (ganglionic eminence, rhombic lip) to mature cortical and other grey matter structures and formation of the cytoskeletal network necessary for mature neuronal connectivity. While our data suggests that neurons in these neurogenic regions express significantly lower levels of tau, confirmation will require more detailed histopathological studies on human tissue sections.

The precise timing of this shift has not previously been reported in humans and raises interesting questions about the normal developmental role of the shift in tau protein splicing and why it is so strongly conserved across multiple species. Tau expression and splicing may also serve as a useful biomarker for neuronal and overall brain maturation. Our findings also reinforce the critical role of developmental stage in determining tau expression in induced pluripotential stem cells (iPSCs) [42]. Interestingly, apart from transient immature areas that involute during fetal development, there is little regional variation in *MAPT* expression or splicing, particularly within different neocortical regions.

Our study is limited by the fact that it is based in part on existing publicly available data sets with variable tissue sampling. Despite this, the replicability of our findings in multiple independent data sets using four different analytic modalities (RNAseq, microarrays, qPCR and western blotting) suggests that the basic conclusions are robust. The study of complex alternative splicing by microarray or even conventional short-read RNA sequencing, as was the case in all studies included here, is also limited in that it does not permit analysis of combinatorial exon utilization at the mRNA level, although this was possible in our western blots. This makes it difficult to assess the abundance of full-length transcripts relative to each other and precludes detailed examination of potential regulatory sequences or retained introns. By definition, all of the specimens, particularly cases of sudden infant death syndrome (SIDS) were also exposed to some degree of terminal hypoxic ischemic injury. While tau splicing is affected in some animal stroke models, the effect of subtler forms of hypoxic injury is unclear. The available data suggests, however, that hypoxic-ischemic injury causes a reversion to fetal tau isoforms, which would, if present, bias the study towards the null hypothesis [43].

Our results suggest that tau undergoes marked changes in splicing during fetal development and demonstrate that this shift is rapid, occurs during the perinatal period and involves predominantly exons 2 and 10. We have also shown that this change in splicing is unique to *MAPT* and is not seen in any of the related human microtubule associated proteins. This study extends the limited existing animal data based on brain homogenates to humans and demonstrates regional variation in tau expression that has not been previously described in humans or animals. Although the functional significance of the developmental switch in tau splicing remains unclear, its strong evolutionary conservation, and the fact that abnormalities in tau splicing have significant implications for axonal transport [44], amyloid processing [45] and the pathogenesis of human tauopathies [46–48] suggests that fetal isoform expression may play a necessary role in development while becoming toxic in the adult brain. Elucidating these functions will require more detailed animal and human studies. At the same time, more detailed studies of combinatorial exon expression, in conjunction with studies of splicing quantitative trait loci and protein-RNA interaction in human development will be needed to better understand the underlying regulatory mechanisms and lead to future insights into the pathogenesis of tau related diseases.
